# Impact of Clinical Examination and Gamma Knife Surgery in Stage IV Breast Cancer With Brain Metastasis

**DOI:** 10.7759/cureus.51831

**Published:** 2024-01-08

**Authors:** Nandan M Shanbhag, Martin C Tom, Albert Duncan, Abdulrahman Bin Sumaida

**Affiliations:** 1 Oncology, Tawam Hospital, Al Ain, ARE; 2 Internal Medicine, United Arab Emirates University, Al Ain, ARE; 3 Radiation Oncology, MD Anderson Cancer Center, Houston, USA; 4 Surgery, Mount St. John’s Medical Center, St. John's, ATG; 5 Oncology, Radiation Oncolgy, Tawam Hospital, Al Ain, ARE

**Keywords:** multidisciplinary treatment, brain metastasis, gamma knife radiosurgery, clinical examination, stage iv breast cancer

## Abstract

Metastatic breast cancer often presents with significant diagnostic and treatment challenges. This case report highlights the crucial role of thorough clinical examination and history-taking in diagnosing and managing a patient with metastatic breast cancer, mainly focusing on the successful integration of Gamma Knife radiosurgery (GKRS). We present a case of a 68-year-old postmenopausal woman with metastatic breast cancer, initially presenting with a primary tumour in the left breast and later developing a solitary brain metastasis (BM) in the left temporal lobe. Following neoadjuvant chemotherapy and left mastectomy, the patient experienced involuntary movements in the right arm, leading to the discovery of the brain lesion. Critical to this diagnosis was a detailed clinical examination emphasising the importance of vigilant monitoring in cancer management. The patient underwent GKRS, offering a focused and less invasive treatment approach with favourable outcomes. This case underscores the value of clinical vigilance in managing complex breast cancer cases. The integration of GKRS as a targeted treatment modality for BM represents a pivotal aspect of modern oncological care, especially for patients with multiple treatment modalities. This report emphasizes the importance of clinical examination in the early detection of complications such as BM in breast cancer patients. Furthermore, it demonstrates the effectiveness of GKRS in managing such metastases, reinforcing its role as a valuable tool in the multidisciplinary treatment approach for advanced breast cancer.

## Introduction

Breast cancer is the most common malignancy in women worldwide and often poses significant challenges in its advanced stages, mainly when it metastasizes to distant organs such as the brain. Brain metastases (BM) have an estimated prevalence of around 9% and occur more frequently in individuals with advanced-stage disease [1]. Additionally, patients with stage III triple receptor-negative breast cancer have a notably higher risk of developing BM as a first site of recurrence, which is associated with poor survival [2]. BM are associated with significant morbidity and mortality, and their treatment is challenged by the blood-brain barrier and the unique microenvironment of the central nervous system [3].

The importance of clinical examination in identifying BM in breast cancer patients is critical, as timely and accurate diagnosis can significantly affect treatment decisions and outcomes. While advanced imaging techniques such as contrast-enhanced computed tomography (CECT) and magnetic resonance imaging (MRI) with contrast are indispensable tools, the initial recognition of potential BM often relies on thorough clinical evaluation [4].

The traditional treatment modalities for BM include whole-brain radiotherapy (WBRT), surgical resection, and stereotactic radiosurgery (SRS), with Gamma Knife radiosurgery (GKRS) being a prominent form of SRS. Recent advances in cancer therapy have improved the survival rates of breast cancer patients. GKRS has emerged as a pivotal treatment modality, offering focused and less invasive intervention for BM. Studies have shown that GKRS provides excellent palliation with a low incidence of toxicity in treating BM in breast cancer patients [5]. It is known for its consistent results with reproducible local tumour control rates of 90-94% for breast cancer metastases [6]. GKRS has been deemed effective and relatively safe even for brain stem metastases, with an overall tumour control rate of 77.4% using a marginal dose of 15 Gy or less [7]. The median overall survival for breast cancer patients treated with GKRS for BM is reported to be around 11 months, indicating a significant extension in survival post-treatment [8]. Additionally, GKRS has been shown to improve local control and survival compared to WBRT alone, with less impact on neurocognitive functions [9].

## Case presentation

A 68-year-old female presented with concerns about a firmness in her left breast that she had self-palpated nine months prior. Despite undergoing multiple mammograms that returned normal (BI-RADS 2), the persistence of a breast skin thickening with a 'peau d' orange appearance was a concern and warranted further investigation. She underwent an ultrasound of the breasts and an incisional biopsy of the left breast lesion. The pathology results revealed a diagnosis of invasive carcinoma, likely of ductal origin, accompanied by high-grade ductal carcinoma in situ and extensive lymphovascular invasion. This was clinically staged as stage IIIB (cT4N1M0), according to the American Joint Committee on Cancer (AJCC) [10].

Her medical history was notable for diabetes mellitus, hypertension, and uterine fibroids, and she had undergone upper back surgery in 1998 for a benign cause. The patient's family history revealed a potential genetic predisposition to cancer, with her father having passed away from lung cancer and her mother from breast cancer. In her social history, she denied any history of smoking or alcohol use and had adequate support from her family. Her performance status was good and deemed as Eastern Cooperative Oncology Group 1 (ECOG) [11].

Following the biopsy, her treatment regimen commenced with four cycles of neo-adjuvant chemotherapy using doxorubicin and cyclophosphamide, completed by February 2021. In March 2021, she underwent a left breast mastectomy with axillary sampling, a definitive step in addressing the localized cancer. Post-mastectomy, the tumour was staged as invasive ductal carcinoma, Grade 2 (ypT2 ypN3a cMx).

However, during her follow-up to start paclitaxel chemotherapy, she reported new symptoms: uncontrolled movement in her right arm and weakness in her right leg. A thorough clinical exam confirmed the signs of involuntary movement in the right arm, prominent and exaggerated when trying to reach for an object (Video 1).

**Video 1:** Involuntary movements in the right upper limb The video demonstrates involuntary movements in the right upper limb due to an 11-mm metastatic lesion in the left hypothalamic region. Patient consent was obtained. Video Credit: Nandan Shanbhag

A brain computed tomography (CT) scan with intravenous contrast revealed a solitary hyperdense lesion in the left subthalamic region, suggestive of metastasis. Further imaging in May 2021, a brain magnetic resonance imaging (MRI), indicated post-mastectomy changes and confirmed an 11 mm enhancing lesion in the left subthalamic region, consistent with metastasis. The PET/CT scan, though, showed no evidence of any recurrent breast malignancy or metastatic disease.

**Figure 1:**
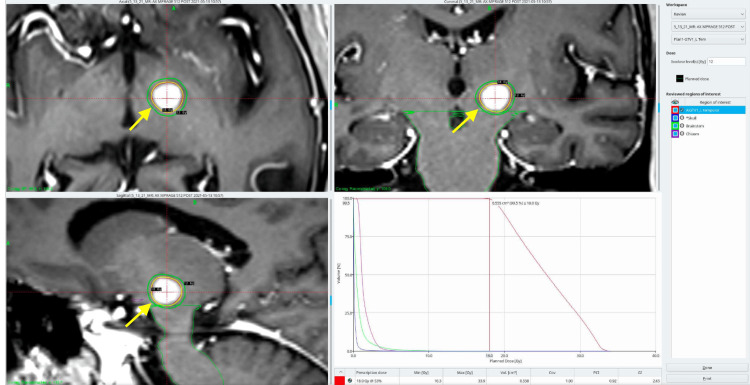
MRI depicting the tumour location with contours and the DVH The figure depicts the tumour's exact location in the three dimensions, and the contours are marked with a yellow arrow. A dose-volume histogram is also presented, demonstrating the doses to the various volumes of the target lesion and the organs at risk. Figure Credit: Martin Tom

She further completed four cycles of paclitaxel chemotherapy. She continued to the next phase of her treatment, which involved GKRS for the solitary brain metastasis in the left temporal lobe, conducted in May 2021. Under local anaesthesia and intravenous sedation, a stereotactic head frame was applied to her skull. The procedure involved fusing cone beam CT (CBCT) images with MRI images and creating an empiric treatment plan, which included a prescription dose of 18 Gy and targeted specific lesion dimensions (Table 1, Figure 1).

**Table 1:** Treatment dimensions and dose prescription The table depicts the target dimensions in centimetres (cm) and the dose prescription and treatment details. mm: millimetres; cc: cubic centimetres

Aantero-posterior	1.0 cm
Lateral	1.06 cm
Vertical	0.96 cm
Prescription Dose = 18Gy	
Prescription Isodose Line	53%
Prescription Isodose Volume	0.63 cc.
12 Gy Volume:	1.08 cc
Number of Isocenters	23
Sector Collimation	4 mm
Treatment Time	68.9 minutes

The treatment, which lasted approximately 68.9 minutes, was carried out without complications, and the patient was returned to observation in stable condition. The patient then completed her post-mastectomy radiation course to the local chest wall and the nodal area.

Follow-up

The patient completed her treatment as planned and was stable on follow-up with no further evidence of disease spread six months from the last day of treatment. Her involuntary movements in the right arm and weakness in the lower limb showed marked improvement.

## Discussion

The prognosis of stage IIIB breast cancer varies and is influenced by several factors, including the presence of metastases, response to chemotherapy, and individual patient characteristics. For instance, the prognosis for patients with ipsilateral supraclavicular lymph node metastasis in advanced breast cancer, while being better than stage IV, was slightly worse than for those with stage IIIb/c breast cancer [12]. Furthermore, there is better overall survival with stage IIIB, palpable breast mass at diagnosis, and favourable response to initial chemotherapy [13]. A study highlighted that the five-year disease-free survival rate for stage IIIB breast cancer patients was 33% following treatment with combination chemotherapy, surgery, and radiation therapy [14]. Another study observed that patients with ipsilateral supraclavicular metastases who received combined-modality therapy had similar clinical outcomes and prognoses as those with stage IB breast cancer [15].

The development of brain metastasis, as observed in this patient, significantly affects the prognosis. A study reported that the incidence of brain metastasis among patients with stage III inflammatory breast cancer was 5%, 9%, and 18%, with triple-negative breast cancer having the worst overall survival (0.2 years) [16]. The survival outlook for breast cancer patients with brain metastases is generally poor, but there are some long-term survivors [17].

Clinical examination plays a crucial role in the early detection and diagnosis of brain metastases in breast cancer patients. Brain metastases occur in a significant portion of breast cancer patients, and early detection is critical for effective management [18]. Involuntary movements, particularly chorea, are significant clinical symptoms that may indicate underlying brain pathologies, including brain metastases. Chorea is characterized by irregular, involuntary movements often accompanied by writhing movements. It can be caused by various disorders, including brain metastases, and presents diagnostic and therapeutic challenges. The presence of chorea in patients, particularly those with a history of cancer, should prompt a thorough investigation for possible brain metastases. Adult-onset chorea can be caused by multiple cerebral metastatic lesions in the basal ganglia and thalamus, a rare but potentially fatal condition, and is recognized as a complex disorder occurring due to various disorders and drug-induced syndromes; it is also one of the most common movement disorders following a stroke, particularly in older patients, often associated with deep vascular lesions, and described as a complex neuronal network disorder caused by dysfunctional neuronal networks [19,20].

Historically, a CT scan was the first imaging modality in a suspected brain space-occupying lesion [21,22]. Brain metastasis incidence is about 20-40% in breast cancer patients, and the contrast-enhanced MRI is the gold standard for their diagnosis [23]. Advanced MRI techniques, including post-gadolinium T1-weighted and T2-weighted FLAIR, have significantly enhanced the management of brain metastases, from early detection to treatment response evaluation [24]. The use of gadolinium MRI of the brain and/or spinal cord, along with cytological examination of cerebrospinal fluid (CSF), is a current diagnostic method for leptomeningeal metastases in breast cancer patients [25]. Advances in neuroimaging and a multidisciplinary approach play a crucial role in managing brain metastases in breast cancer patients; several factors affect survival in these patients, such as performance status, number of brain metastases, treatment modalities, and systemic chemotherapy [26].

In the case presented, the patient underwent neo-adjuvant chemotherapy, mastectomy, and GKRS for brain metastasis. SRS has become a vital tool in treating brain metastases and is known for its effectiveness and safety. Notably, research from the early 1990s identified SRS as highly effective, achieving substantial tumour control and complete response rates [27]. It is particularly beneficial for small, multiple, and deep brain metastases, often used alongside whole-brain radiation therapy to improve control. Studies have shown its capability in managing intracranial disease with high local control rates in patients with 10 or more brain metastases [28]. Moreover, SRS significantly improves survival rates, particularly in newly diagnosed multiple intracranial metastases [29]. Technological advances, such as the CyberKnife system, have further enhanced the efficacy of SRS in both benign and malignant brain tumours [30,31]. Compared to other therapies, SRS is a comparable alternative for small, single-brain metastases and is often combined with whole-brain radiation in cases of multiple metastases [32].

The safety profile of SRS is another significant advantage. It is a less invasive option, reducing the risks associated with surgical procedures such as craniotomy and resection and making it a safer choice for certain patients [33]. SRS represents a transformative approach to treating brain metastases, blending efficacy, safety, and versatility, as evidenced in various studies and clinical trials.

## Conclusions

The management of brain metastases in breast cancer requires a multidisciplinary approach, integrating surgical, systemic, and radiation therapies. Advancing neuroimaging techniques has enhanced the ability to detect and monitor these metastases, contributing to more personalized and effective treatment strategies.

Overall, the case highlights the importance of a comprehensive approach in managing advanced breast cancer, considering the individual patient's disease characteristics, response to treatment, and evolving therapeutic options. This approach is essential for improving survival rates and the quality of life for breast cancer patients with brain metastases.
